# Hypertensive Disorders of Pregnancy and Peripartum Cardiomyopathy: A Meta-Analysis of Prevalence and Impact on Left Ventricular Function and Mortality

**DOI:** 10.3390/jcm14051721

**Published:** 2025-03-04

**Authors:** Aleksandar Biljic-Erski, Nina Rajovic, Vedrana Pavlovic, Zoran Bukumiric, Aleksandar Rakic, Marija Rovcanin, Jelena Stulic, Radomir Anicic, Jovana Kocic, Jelena Cumic, Ksenija Markovic, Dimitrije Zdravkovic, Dejana Stanisavljevic, Srdjan Masic, Natasa Milic, Dejan Dimitrijevic

**Affiliations:** 1 Institute for Medical Statistics and Informatics, Faculty of Medicine, University of Belgrade, 11030 Belgrade, Serbia; 2 Department of Gynecology and Obstetrics, University Hospital Center “Narodni Front”, 11030 Belgrade, Serbia; 3 Faculty of Medicine, University of Belgrade, 11030 Belgrade, Serbia; 4 Clinic for Cardiac Surgery, Department of Anesthesiology, Clinical Centre of Serbia, 11030 Belgrade, Serbia; 5 Department of Primary Health Care and Public Health, Faculty of Medicine, University of East Sarajevo, 73300 Foca, Bosnia and Herzegovina; 6 Division of Nephrology and Hypertension, Mayo Clinic, Rochester, MN 55905, USA

**Keywords:** peripartum cardiomyopathy, preeclampsia, meta-analysis, hypertensive disorder, pregnancy

## Abstract

**Background:** The purpose of this meta-analysis was to examine the prevalence of hypertensive disorders of pregnancy (HDPs), particularly preeclampsia (PE), in peripartum cardiomyopathy (PPCM)-affected pregnancies, and to evaluate whether a HDP significantly alters the prognosis of PPCM, with specific reference to the recovery of left ventricular function (LVEF) and mortality. **Methods:** A total of 5468 potentially eligible studies were identified, and 104 were included in the meta-analysis. For pooling proportions, the inverse variance methods with logit transformation were used. Complete recovery of LVEF (>50%) and mortality were expressed by odds ratios (ORs), with 95% confidence intervals (CIs). The Peto OR (POR) was used in cases of rare events. Baseline LV function and baseline LV end-diastolic diameter (LVEDD) were summarized by the mean difference (MD) and 95% confidence interval (CI). **Results:** The summary estimate of the prevalence of HDPs and PE in women with PPCM was 36% and 25%, respectively. Patients with HDPs and, more specifically, PE with PPCM had a higher chance of complete recovery (OR = 1.87; 95%CI = 1.64 to 2.13; *p* < 0.001 and OR = 1.98; 95%CI 1.69 to 2.32; *p* < 0.001, respectively), a higher baseline LVEF (MD, 1.42; 95% CI 0.16 to 2.67; *p* = 0.03 and MD, 1.69; 95% CI 0.21 to 3.18; *p* = 0.03, respectively), and a smaller baseline LVEDD (MD, −1.31; 95% CI −2.50 to −0.13; *p* = 0.03 and MD, −2.63; 95% CI −3.75 to −1.51; *p* < 0.001, respectively). These results, however, did not translate into a significant difference in 12-month mortality (POR = 0.80; 95% CI = 0.57 to 1.13; *p* = 0.21 and POR = 1.56; 95% CI 0.90 to 2.73; *p* = 0.12, respectively). **Conclusions:** The findings of this study may contribute to evidence that can be utilized to aid in the risk stratification of patients with PPCM regarding their long-term prognoses.

## 1. Introduction

Peripartum cardiomyopathy (PPCM) is a condition presenting with systolic heart failure (HF) with reduced left ventricular ejection fraction (LVEF) toward the end of pregnancy or in the months following delivery. It is a diagnosis of exclusion in peripartum women when no other cause is identifiable [1]. There have been several prior definitions of PPCM that have included symptomatic HF occurring in the last month of pregnancy and up to 5 months postpartum [2,3]. Definitions have changed over time, and the European Society of Cardiology (ESC) position paper in 2019 described PPCM as “an idiopathic cardiomyopathy presenting with HF secondary to left ventricular (LV) systolic dysfunction, with a LVEF < 45% towards the end of pregnancy, or in the months following delivery, where no other cause of heart failure is identified” [4]. Peripartum cardiomyopathy affects approximately 1 in 2000 births globally [5], showing significant regional variation, with incidence rates reaching as high as 1 in 300 births in Haiti [6] and 1 in 100 in Nigeria [7]. The reported incidence of PPCM in the United States varies between 1 in 1000 and 1 in 2000, as documented in previous studies [8,9]. This prevalence may be on the rise due to factors such as advanced maternal age, higher rates of multi-fetal pregnancies resulting from modern fertility procedures, and improved awareness and detection of the disease. There is a possibility that numerous incidents may go unnoticed, resulting in an unknown true occurrence rate [1]. Given its diverse clinical presentations and yet unknown etiology, numerous hypotheses have been proposed related to the risk factors which may contribute to its development. Preeclampsia (PE) with pulmonary edema is a common condition in which PPCM can be identified, and according to the findings of the meta-analysis conducted by Bello et al. [10], the prevalence of PE was 22% among patients with PPCM, compared to the approximately 7.5% background rate [11]. A substantial overlap between the risk factors for PE and PPCM exists. Both are more common in women with prior history of the disease, obesity, multiple pregnancies, diabetes mellitus, and advanced reproductive age [1,12]. It is reasonable to hypothesize that there may be a common pathophysiologic link between these two phenomena. A recent study implicated the process of senescence, one of the key mechanisms of aging, and the senescence-associated secretory phenotype (SASP), derived from the accelerated placental senescence, in the pathophysiology of PPCM, particularly in the context of PE [13]. However, there is still ongoing debate as to whether patients with hypertensive diseases of pregnancy (HDPs) like PE should be considered to have PPCM [14]. While one group of authors describes the protective effect of HDPs on PPCM outcomes [15,16,17], others have found no differences in outcomes of PPCM, with or without subsequent PE [18,19]. Improved recovery of HDP-related cardiomyopathy has been posited to be, in part, an effect of earlier presentation due to symptomatic hypertension or pulmonary edema in HDPs, less so protective from the HDP itself [20]. The ESC Peripartum Cardiomyopathy Registry has observed distinct variations among women diagnosed with PPCM based on the presence or absence of HDPs. Despite having superior baseline cardiac function, women diagnosed with PPCM and PE presented with more severe symptoms and more frequent signs of HF compared to those without hypertension, but also demonstrated a higher chance of left ventricular recovery. In contrast to recovery of the mother, PPCM and PE was associated with more frequent neonatal death and other adverse neonatal outcomes [17]. Given the dichotomy of these results, further studies are needed to emphasize the relevant maternal morbidity, as well as neonatal outcomes. According to a recently published meta-analysis by Nugrahani et al., PPCM and HPD-PPCM have distinct clinical profiles and forms of remodeling, perhaps influencing their respective responses to pharmaceutical interventions [21]. However, the absence of an evaluation of the prognosis linked to the diagnosis of HDPs in patients with PPCM using echocardiographic findings in the meta-analysis conducted by Bello et al. and Nugrahani et al. makes this specific aspect of the present meta-analysis unique [10,21]. Our objective was to investigate the occurrence of HDPs and PE in pregnancies affected by PPCM and to assess whether the presence of HDPs in PPCM patients had a significant impact on prognosis, specifically in terms of recovery of left ventricular function and mortality rates.

## 2. Materials and Methods

### 2.1. Search Strategy and Selection Criteria

This systematic review was undertaken in accordance with the Preferred Reporting Items for Systematic Reviews and Meta Analysis of Observational Studies in Epidemiology [22,23]. The search strategy was developed by a biostatistician with expertise in conducting systematic reviews and meta-analyses and a clinician (N.M. and D.D.). The search strategy was applied to PubMed, Scopus, Web of Science, and Cochrane Library databases using a date range up until 19 January 2022, in order to retrieve studies containing the keyword, peripartum cardiomyopathy. The search was restricted to articles and abstracts written in the English language. This systematic review and meta-analysis were performed in accordance with PRISMA guidelines.

Two reviewers (A.B.E. and N.R.) independently screened eligible publications in two phases, with all disagreements being resolved by discussion at each stage, or with the adjudication of a third reviewer when consensus could not be established. Studies were eligible for inclusion based on the following criteria: (1) the study included patients with diagnoses of both PPCM and HDP/PE, (2) the study must have had two or more groups, with one of them having patients with HDP/PE, (3) studies with reported sample sizes and numbers of cases, or the prevalence of HDP/PE, (4) studies investigating baseline LVEF and baseline left ventricular end-diastolic diameter, (5) studies investigating at least one of the outcomes of interest, mortality and complete recovery of LVEF (>50%), and (6) original articles. Studies were excluded if they examined other populations or outcomes, were non-English studies, or were conference abstracts, editorials, letters to the editor, case reports, theses, chapters of a book, or reviews. Upon completing the initial screening of an abstract and title, full-text screening was performed independently by each reviewer. If there were any disagreements, the third reviewer independently assessed the articles and provided a final decision as to article inclusion or exclusion. Hypertensive disorders of pregnancy encompassed cases of preeclampsia, eclampsia, chronic hypertension, gestational hypertension, and preeclampsia superimposed on chronic hypertension. A separate analysis included cases of preeclampsia only.

### 2.2. Data Abstraction and Quality Assessment

The following data, study title, author(s), publication year, country in which the study was performed, total number of patients involved, patient ages, rates of HDP, PE, twin and multiple gestations, and multiparity during pregnancy in women with PPCM, were abstracted independently by the reviewers. If any data were missing, the authors of the relevant articles were contacted. The quality of eligible publications was evaluated separately by each reviewer, using the adapted version of the Newcastle–Ottawa tool for observational studies and the guidelines outlined by the GRADE (Grading of Recommendations, Assessment, Development, and Evaluations) Working Group [24].

### 2.3. Statistical Analysis

Visualizations and prevalence estimates were performed using R “meta”, “metafor”, and “dmetar” packages, Version 4.0.0 (R Core Team 2020: R:A language and environment for statistical computing, R Foundation for Statistical Computing, Vienna, Austria). For pooling proportions, the inverse variance methods with logit transformation were used. Confidence intervals for individual studies were estimated using the Clopper–Pearson method. The heterogeneity was assessed by Cochran’s Q test, Baujat plots, and GOSH graphical displays of heterogeneity, and quantified using I^2^ statistics. Between-group analysis was performed by Review Manager Version 5.4 (Cochrane, 2021). Dichotomous variables, such as complete recovery of LVEF (>50%) and mortality, were expressed by odds ratios (ORs), with their respective 95% confidence intervals (CIs). The Peto OR was used in case of rare events. Continuous variables, such as baseline LV function and baseline LVEDD, were summarized by the mean difference (MD) and 95% CI. Where mean was reported with range, the standard deviation was calculated as (max-min)/4. Heterogeneity was assessed by using Cochran’s Q test and I^2^ statistics. According to the Cochrane Handbook [25], I^2^ < 30%, I^2^ = 30–60%, and I^2^ > 60 correspond to low, moderate, and high heterogeneity of the included publications, respectively. When I^2^ was low or moderate, a fixed-effects model was used, but when I^2^ was high, a random-effects analysis was selected. A separate forest plot was created for each analysis, showing the OR or SMD (box), 95% CI (lines), and weight (size of box) for each publication. A diamond represented the overall effect size. In addition, the same analyses were performed for studies where patients with PE were included. A *p* value of <0.05 was considered to be statistically significant for all analyses. Sensitivity analyses were conducted to examine the effects of studies that included large cohorts of women with PPCM.

## 3. Results

### 3.1. Search Results

**Figure 1:**
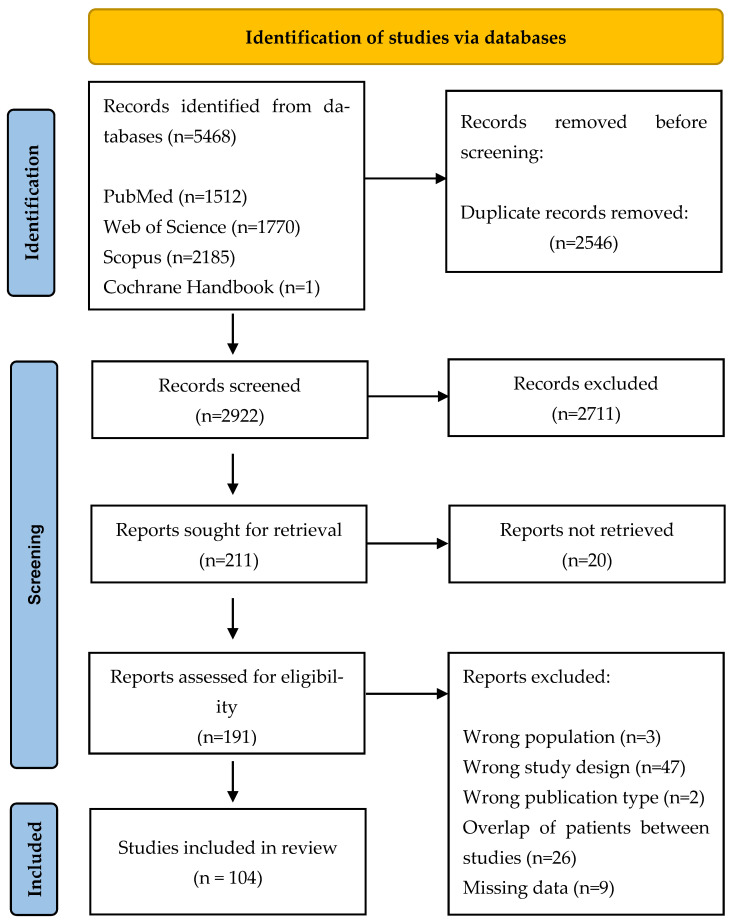
Flowchart of study selection process.

A total of 5468 potentially eligible studies were identified using the search strategy and they were extracted from four electronic databases. When duplicates were removed, 2922 abstracts were screened for eligibility. Finally, 2731 studies did not meet the criteria for inclusion, and a total of 191 were assessed for eligibility. After evaluating the full texts, 47 studies were excluded due to wrong study design, 26 because of the overlap of patients between studies, 3 because of inclusion of a wrong population, 2 because of the wrong publication type, and 9 because of missing data. Figure 1 presents the study selection process using the PRISMA flow diagram. The quality assessment of selected publications is presented in Supplementary Materials Table S1.

### 3.2. Characteristics of Eligible Studies

A total of 29 eligible studies were conducted in Asian countries, 19 in Europe, 4 in African countries, 48 in American countries, and 1 in Australia, and 3 were multinational studies. The studies were published between 1986 and 2021, and the total number of patients included was 55,714, with sample sizes varying from 5 to 34,219. The minimum average age of patients included was 25 years, and the majority were multiparous. Table 1 describes detailed characteristics of the studies included in the analysis.

### 3.3. Prevalence of HDPs and PE in PPCM-Affected Pregnancies

**Figure 2:**
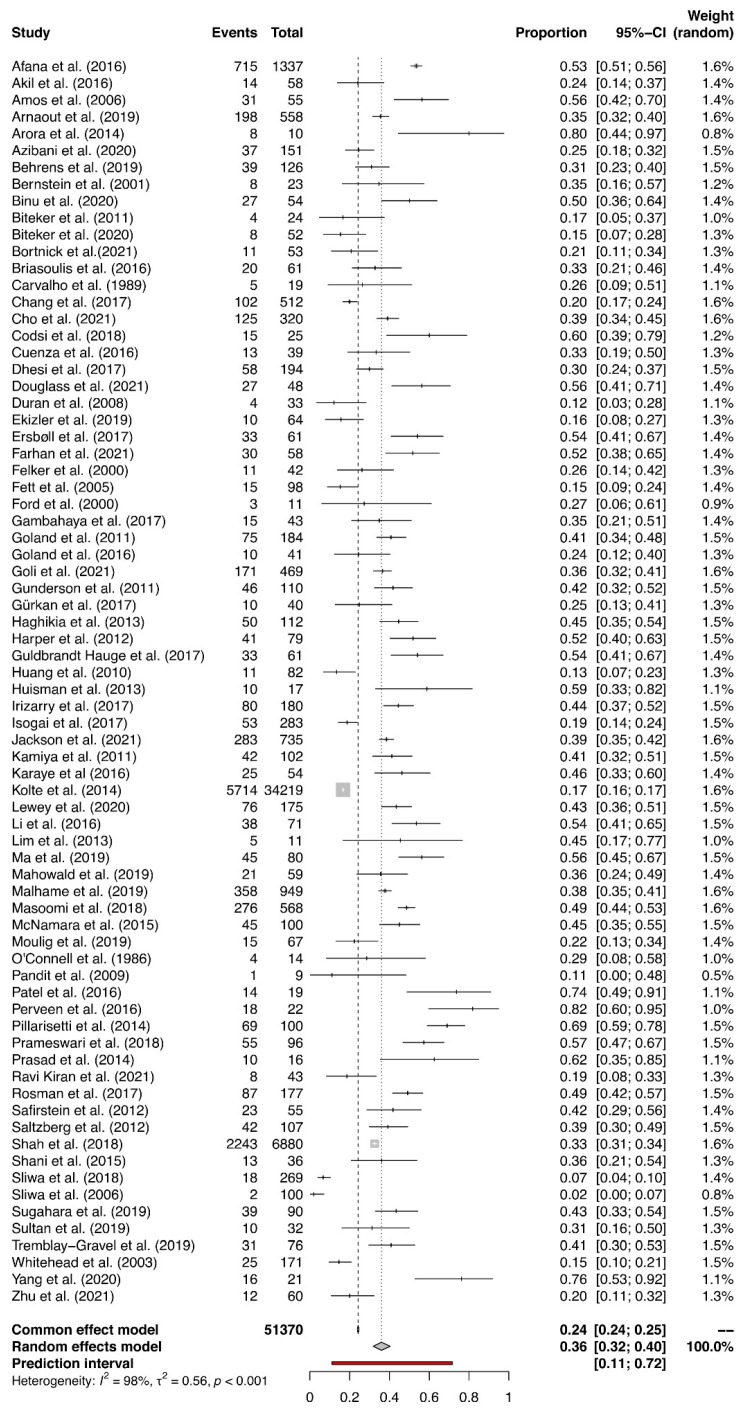
Prevalence estimates for HDPs in women with PPCM.

**Figure 3:**
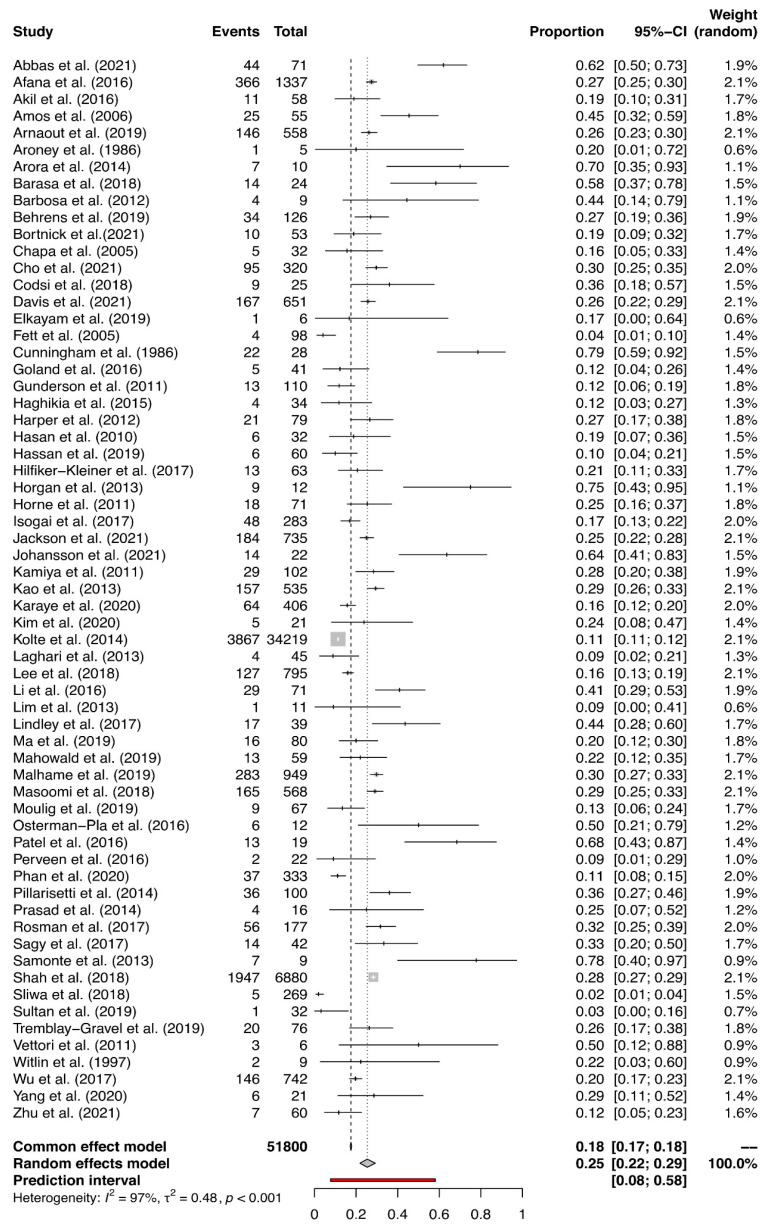
Prevalence estimates for PE in women with PPCM.

The overall prevalence of HDPs varied from a low of 2% to a high of 82% in the individual studies (Figure 2). The summary estimate of the prevalence of HDPs in women with PPCM was 36% (95% CI: 32 to 40), which is more than 2 times higher than the 15.3% worldwide prevalence of HDPs [12]. A high heterogeneity among the included studies (I2 = 98%, χ^2^ = 0.5596, *p* < 0.001) was detected. The estimated overall prevalence of PE in women with PPCM ranged from 2% to 79% in the individual studies (Figure 3). The summary estimate of 25% was almost 3 times higher than the 7.5% worldwide prevalence of PE [11]. There was substantial heterogeneity among the included studies (I^2^ = 97%, χ^2^ = 0.4823, *p* < 0.001). Although influence analysis and Baujat graphs detected one study [82] as influential, sensitivity analysis and GOSH graphs presented no effect of this study on the pooled prevalence estimate (Supplementary Materials Figure S1a,b).

### 3.4. Results of the Meta-Analysis

#### 3.4.1. Baseline LVEF for HDPs and PE in Women with PPCM

**Figure 4:**
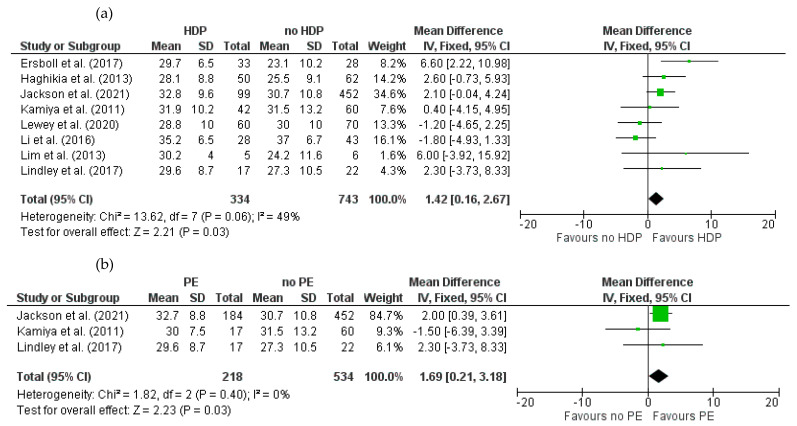
Forest plot comparing the baseline left ventricular ejection fraction (LVEF) between patients with and without (**a**) HDPs and (**b**) PE.

Baseline LVEF was assessed in eight studies [17,20,56,66,79,85,86,87] with a total of 1077 cases reporting HDP status in PPCM patients (HDPs = 334, no HDPs = 743). There was moderate heterogeneity among studies (I^2^ = 49%). The meta-analysis showed a significantly higher baseline LVEF in HDP patients compared to those without HDPs (MD = 1.42; 95% CI = 0.16 to 2.67; *p* = 0.03) (Figure 4a).

Three studies [18,79,87] with a combined total of 752 cases reported baseline LVEF according to PE status (PE = 218, no PE = 534). There was no heterogeneity among studies (I^2^ = 0%). The meta-analysis showed that PE patients had a significantly higher LVEF versus those without PE (MD = 1.69; 95% CI = 0.21 to 3.18; *p* = 0.03) (Figure 4b).

#### 3.4.2. Baseline LVEDD for HDPs and PE in Women with PPCM

**Figure 5:**
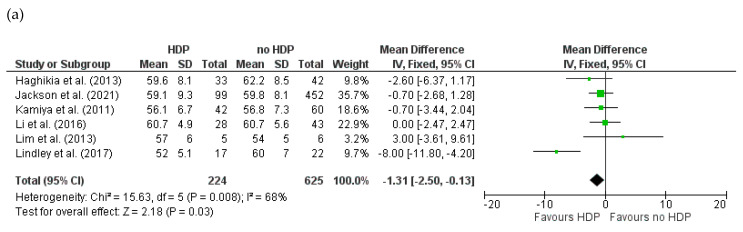

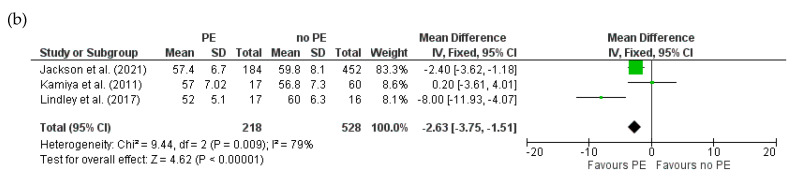
Forest plot comparing the baseline left ventricular end-diastolic diameter (LVEDD) between women with and without (**a**) HDPs and (**b**) PE.

Baseline LVEDD was assessed in six studies [17,66,79,85,86,87] comprising 849 cases reporting HDP status in PPCM patients (HDPs = 224, no HDPs = 625). There was high heterogeneity among the studies (I^2^ = 68%). The meta-analysis indicated significant differences in baseline LVEDD between patients with and without HDPs. HDP patients had a significantly lower baseline LVEDD compared to patients without HDPs (MD = −1.31; 95% CI = −2.50 to −0.13; *p* = 0.03) (Figure 5a).

Three studies [17,79,86] comprising 746 cases reported baseline LVEDD according to PE status (PE = 218, no PE = 528). There was no heterogeneity among the studies (I^2^ = 0%). The meta-analysis indicated a significant difference in baseline LVEDD between the women with and without PE. PE patients had significantly smaller baseline LVEDD in contrast to women without PE (MD = −2.63; 95% CI = −3.75 to −1.51; *p* < 0.001) (Figure 5b).

#### 3.4.3. Mortality for HDPs and PE in Women with PPCM

**Figure 6:**
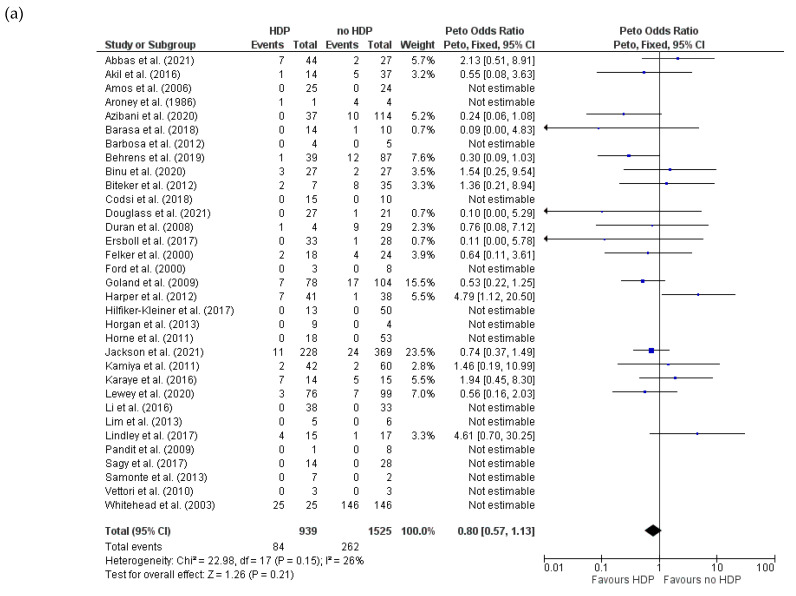

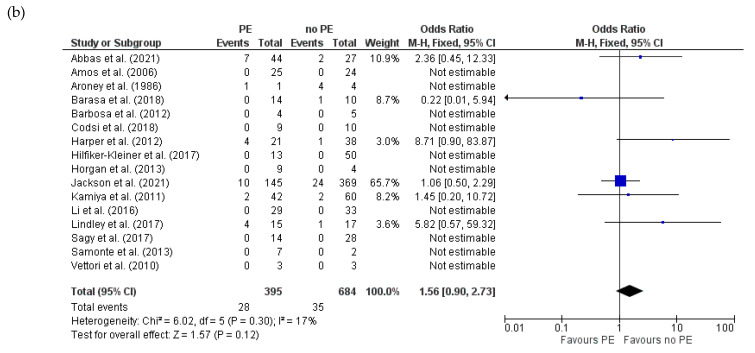
Forest plot comparing mortality between women with and without (**a**) HDPs and (**b**) PE.

Mortality was reported in 33 studies [16,17,20,26,28,29,31,33,34,35,37,38,46,52,53,56,58,59,61,68,71,72,73,79,81,85,86,87,97,106,108,116,117], with a total of 2464 PPCM cases according to HDP status (HDPs = 939, no HDPs = 1525). There was low heterogeneity among studies (I^2^ = 26%). The results of the meta-analysis showed no significant differences in mortality between patients with and without HDPs (POR = 0.80; 95% CI = 0.57 to 1.13; *p* = 0.21) (Figure 6a).

Sixteen studies [16,17,26,29,31,34,46,68,71,72,79,85,87,106,108,116] with a total of 1079 PPCM cases reported mortality according to PE status (PE = 395, no PE = 684). There was low heterogeneity among studies (I^2^ = 17%). The results of the meta-analysis showed no significant differences in mortality between women with and without PE (OR = 1.56; 95% CI = 0.90 to 2.73; *p* = 0.12) (Figure 6b).

#### 3.4.4. Recovery of LVEF (>50%) for HDPs and PE in Women with PPCM

**Figure 7:**
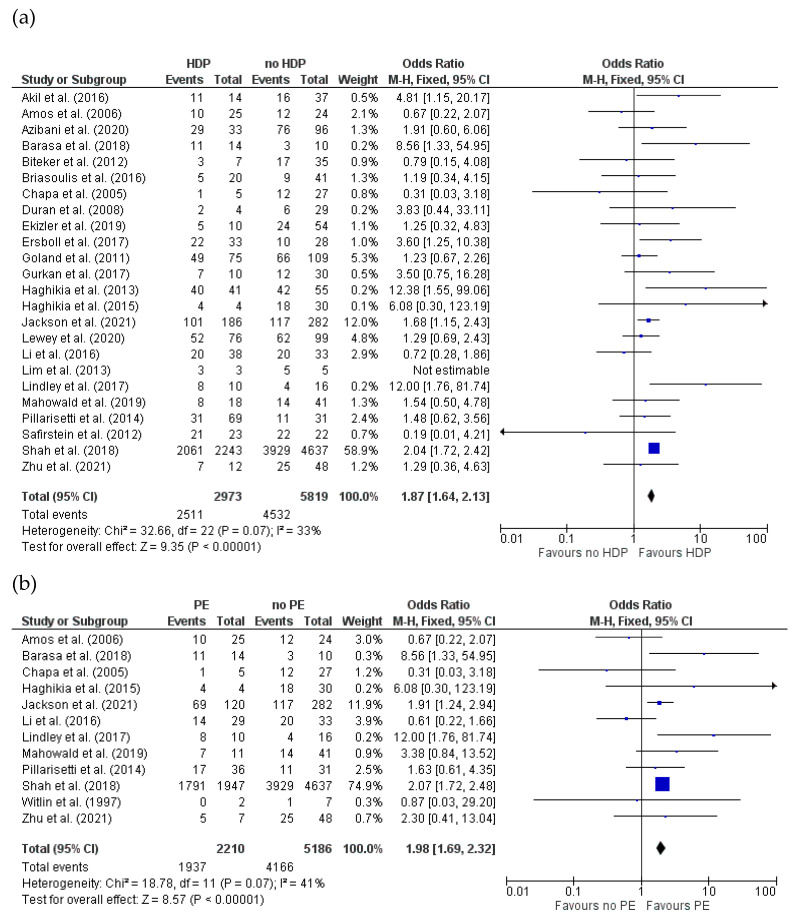
Forest plot comparing the complete recovery of LVEF (>50%) between women with and without (**a**) HDPs and (**b**) PE (Shah et al. 2018 reported data for readmission).

Recovery of LVEF (>50%) was assessed in 24 studies [16,17,19,20,28,29,33,38,41,44,53,54,56,61,65,66,67,85,86,87,89,105,109,121] comprising 8792 cases reporting HDP status in PPCM patients (HDPs = 2973, no HDPs = 5819). The minimum follow-up time was 4 months. Most studies reported 12 months of recovery after LVEF assessment. I^2^ indicated the low heterogeneity among studies (I^2^ = 33%). The results of the meta-analysis showed a significant difference in complete recovery of LVEF (>50%) between patients with and without HDPs. Patients in the HDP group had 1.87-fold greater odds of recovery compared to patients without HDPs (OR = 1.87; 95%CI = 1.64 to 2.13; *p* < 0.00001) (Figure 7a).

Twelve studies [16,17,19,29,44,67,85,87,89,109,118,121] comprising a total of 7396 PPCM cases reported the recovery of LVEF (>50%) according to PE status (PE = 2210, no PE = 5186). Minimum follow-up time was 6 months. Most studies reported 12 months of recovery after LVEF assessment. Moderate heterogeneity among studies was found (I^2^ = 41%). The results of the meta-analysis demonstrated a significant difference in complete recovery of LVEF (>50%) at 12 months between patients with and without PE. PE patients had 1.98-fold greater odds of complete recovery of LVEF compared to patients without PE (OR = 1.98; 95%CI = 1.69 to 2.32; *p* < 0.001) (Figure 7b).

## 4. Discussion

Our present study reports several novel findings regarding the association between HDP/PE and PPCM. First, our analysis found that patients with either HDPs or PE and PPCM, compared to patients with PPCM only, had a higher baseline LVEF and a lower baseline LVEDD. This may suggest that in patients with PPCM only, their less favorable baseline echocardiographic profiles might have contributed to cardiac dysfunction due to hemodynamic and volume challenges of normal pregnancies. Second, in the context of PPCM, we analyzed both HDPs and PE, in contrast to previous meta-analyses, that reported either PE [10] or HDPs [21]. The summary estimate of HDP prevalence in women with PPCM was more than two times higher, while the total prevalence of PE in women with PPCM was almost three times higher than the global rate estimated for the general population. These findings underscore the role of hypertension, which is present in all HDP states, as the driving insult, and not only the systemic endothelial dysfunction, inflammation, and anti-angiogenic states, which are predominantly present in PE. However, the rate of PE seems to be even higher than those of HDPs in women with PPCM, suggesting that vascular and metabolic abnormalities may be shared between PPCM and PE, thus leading to the higher PPCM rates in women with PE. Third, patients with either HDP, or PE and PPM, compared to patients with PPCM only, had higher rates of complete LVEF recovery. This is likely due to (i) better baseline cardiac function and (ii) the largely reversible nature of HDPs (with the exception of chronic hypertension). Fourth, these results, however, did not translate into a significant difference in mortality at 12-month follow-up.

Different studies have assessed the prevalence of hypertensive states in PPCM, that ranged from 2 to 68% [112,118,122]. Results of a systematic review and meta-analysis conducted by Bello et al. [10] in 2013 focused on PE only and demonstrated the variability of PE prevalence in women with PPCM, ranging from 0% to 78%, with an overall summary prevalence of 22%. Similar results were obtained in our study, ten years after, where PE was present in 25% of women with PPCM, with rates ranging from 2% to 79% in the individual studies. Kamiya et al. [79] performed a nationwide survey regarding PPCM and hypertensive disorders complicating pregnancy in Asian countries and noted that the incidence of hypertensive states in PPCM was 41%, which is comparable to the rates of 43% for hypertensive disorders complicating pregnancy found in the study by Elkayam et al. [123], 46% for hypertension found in the study by Modi et al. [124], and to the rate of 22% for preeclamptic patients found in the study by Demakis et al. [2]. Kamiya et al. showed that with maternal age, the frequency of PPCM per 100,000 births increased, notably in the HDP cohort, and this incidence was more than 10 times higher in the group of patients from 35 to 39 years of age. The summary estimates of the prevalence of HDPs and PE in women with PPCM in our meta-analysis are 36% and 25%, respectively, and similar to those reported previously.

Preeclampsia has long been regarded as a risk factor for PPCM, and a large body of evidence has suggested that PPCM risk may depend on the severity of HDPs [8,80,125]. In keeping with this, we report that rates of PE are even higher than those of HDPs in women with PPCM. In a recently published nationwide study performed by Behrens et al. [35], in which more than 2 million pregnancies were included in the cohort, it was demonstrated that HDPs were significantly associated with PPCM, and that the risk of PPCM substantially increased from 5 to 21 times in women with HDPs compared to normotensive women during pregnancy. Rapid recovery of LVEF has been observed in patients with concomitant PE, most recently by Ntusi et al. [18], who discovered that PPCM with any hypertensive HF, compared to PPCM only, had substantially reduced mortality during the 14 years of follow-up. Of note, our meta-analysis showed no difference in mortality, but analysis was limited to the follow-up period of only 12 months. A study of patients with PPCM from five hospitals in Sweden was published by Barasa et al. [16] in 2017, and the results demonstrated that women with concomitant PE were more likely to develop acute pulmonary edema than those without; however, they recovered more rapidly and had a better prognosis. Barasa et al. [16] additionally showed that a higher LVEF was seen in PE, compared to PPCM patients without PE, both initially and throughout the follow-up. Similar conclusions were recently reached by Kamiya and colleagues [79], who compared the clinical profiles of Japanese PPCM patients with and without gestational hypertension and discovered that those with pregnancy-associated hypertension had shorter hospital stays and a higher LVEF at the time of their last follow-up. These reports are in agreement with our meta-analysis results that included studies that were performed globally.

Elevated risk for PPCM was observed among women of African descent in many studies [27,126,127]. Although the Black community accounts for less than 15% of the total US population, in two recent studies, Black women constituted nearly half of PPCM cases [8,128]. The incidence of PPCM was found to be three to four times greater in Black women compared to White women, whereas the lowest incidence was observed in Hispanic women [8,68,80,129]. Due to the diversity of results conducted thus far and the lack of data from Africa and the Caribbean, it remains uncertain whether there are disparities in the correlation between PPCM and PE among Black women, who exhibit elevated prevalence rates of both conditions, compared to women of other racial and ethnic backgrounds [10]. Notably, a study of an African American cohort [87] demonstrated that PPCM patients with PE had significantly worse one-year morbidity and mortality, which is in contrast to our results. Differences in outcomes compared to other studies and our current meta-analysis might be explained by genetic differences, lifestyle, and disparities in medical standards and health care access and need to be investigated further.

Despite several proposed mechanisms, the nature of the association between HDP/PE and PPCM is still unclear. The heterogeneity of PE is increasingly recognized, and it is likely that different clinical presentations are results of different underlying mechanisms. Along those lines, HDP/PE and PPCM may result from a pathophysiological process, such as senescence (as suggested recently) [13], that starts in pregnancy and persists after delivery. Considering that cardiovascular diseases, including cardiomyopathy, are among the primary causes of pregnancy-related deaths, rapid identification and early management of women at the highest risk of cardiovascular morbidity are critical steps to reduce maternal mortality. The mainstay of therapy at present is treatment of gestational and chronic hypertension during pregnancy to blood pressure < 140/90 mmHg, according to the guidelines from the American College of Obstetrics and Gynecology.

Our study has several limitations. There is a considerable heterogeneity of studies performed to date, and data stratified by race, age, and region, which could provide valuable insights, are limited. In addition, our results demonstrate the lack of correlation between the protective role of HDPs in PPCM recovery and mortality; however, the sample size was insufficient and a follow-up of 12 months was inadequate to establish a definitive conclusion. Despite these limitations, our meta-analysis is the first to evaluate the prognostic implications associated with the diagnosis of HDP/PE in patients diagnosed with PPCM utilizing echocardiographic data, such as baseline LVEF and LVEDD, and specifically focusing on complete LVEF recovery and 12-month mortality. Future research, aimed at improving understanding of risk factors and, even more importantly, the underlying mechanism(s) that are common to HDP/PE and PPCM, is needed. This may allow for screening and timely recognition of women at risk and, ultimately, targeted therapies.

## 5. Conclusions

Our study highlights important associations between HDP/PE and PPCM. The prevalence of HDPs and PE in PPCM patients was 36% and 25%, respectively. Patients with HDPs or PE and PPCM have better baseline cardiac function and higher rates of LVEF recovery compared to those with PPCM alone, likely due to the reversible nature of HDPs. Despite these findings, there was no significant difference in mortality at 12 months, underscoring the complexity of these conditions and the need for further research. The findings of this study may contribute to evidence that can be utilized to aid in the risk stratification of patients with PPCM regarding their long-term prognoses.

## Figures and Tables

**Table 1:** Characteristics of studies included in the meta-analysis.

Study	Year	Country	No. of Patients	Age (yrs), Mean ± sd	PE (%)	HDP (%)	Twins or Multiples (%)	Multiparous, n (%) or Mean ± sd
Abbas et al. [26]	2021	Pakistan	71	29.8 ± 6.8	44 (62)	NA	NA	57 (80.3)
Afana et al. [27]	2016	USA NIS database	1337	35+, n (%): 314 (23.5)	366 (27.4)	715 (53.5)	96 (7.2)	
Akil et al. [28]	2016	Turkey	58	31.5 ± 6.3	11 (21.6)	14 (27.5)	7 (13.7)	38 (73.1)
Amos et al. [29]	2006	USA	55	29 ± 6	25 (46)	31 (56)	NA	NA
Arnaout et al. [30]	2019	USA	558	NA	146 (26.1)	198 (35.4)	NA	NA
Aroney et al. [31]	1986	Australia	5	28.8 ± 5.5	1 (20)	NA	2 (40)	4 (80)
Arora et al. [32]	2014	USA	10	28 ± 6	7 (70)	8 (80)	1 (10)	8 (80)
Azibani et al. [33]	2020	Germany and S. Africa	151	31 ± 6	NA	37 (26)	NA	NA
Barasa et al. [16]	2018	Sweden	24	34.2 ± 5.0	14 (58.3)	NA	5 (20.8)	1.6 ± 0.7
Barbosa et al. [34]	2012	Brazil	9	29.7 ± 7.9	4 (44)	NA	NA	NA
Behrens et al. [35]	2019	Denmark	126	35+, n (%): 37 (29.4)	34 (27)	39 (31.0)	10 (7.9)	69 (54.8)
Bernstein et al. [36]	2001	USA	23	30.9 ± 4.6	NA	8 (35)	4 (17)	16 (70)
Binu et al. [37]	2020	India	54	25.5	NA	27 (50)	7 (13.0)	29 (35.2)
Biteker et al. [38]	2011	Turkey	24	26.6 ± 5.5	NA	4 (16.7)	2 (8.3)	1.7 ± 0.8
Biteker et al. [39]	2020	Turkey	52	28.0 ± 5.3	NA	8 (15.4)	2 (3.8)	2.6 ± 1.0
Bortnick et al. [40]	2021	USA	53	31 ± 7	10 (18.9)	11 (20.8)	NA	2 ± 1.3
Briasoulis et al. [41]	2016	USA	61	29	NA	20 (32.8)	NA	45 (73.7)
Carvalho et al. [42]	1989	USA	19	25.9 ± 7.2	NA	5 (26.3)	NA	10 (52.6)
Chang et al. [43]	2017	Taiwan	512	35+, n (%): 93 (18.16)	NA	102 (19.92)	38 (7.42)	NA
Chapa et al. [44]	2005	USA	32	27 ± 6	5 (15.6)	NA	4 (12.5)	Median (range): 2 (1–6)
Cho et al. [45]	2021	USA	320	30.5 ± 7.0	95 (29.7)	125 (39.1)	34 (10.6)	177 (55.3)
Codsi et al. [46]	2018	USA	25	Median (range): 26 (15–37)	9 (36.0)	15 (60)	4 (16.0)	Median (range): 0 (0–3)
Cuenza et al. [47]	2016	Philippines	39	28.4 ± 6.9	NA	13 (33)	1 (0.25)	16 (41)
Cunningham et al. [48]	1986	USA	28	28.5 ± 6.5	22 (78.6)	NA	NA	2.2 ± 2.3
Davis et al. [49]	2019	USA	100	30 ± 6	NA	45 (45)	19 (19.0)	Median (range): 2 (1–6) (1–6)
Davis et al. [50]	2021	USA	651	30+, n (%): 299 (45.9)	167 (26)	NA	70 (11)	NA
Dhesi et al. [51]	2017	Canada database	194	30.4 ± 6.6	NA	58 (29.9)	16 (8.2)	76 (39.2)
Douglass et al. [52]	2021	USA	48	28 ± 7	NA	27 (56.3)	8 (17)	Median (range): 1 (0–2.5)
Duran et al. [53]	2008	Turkey	33	32 ± 7	NA	4 (12.1)	2 (6)	Median (range): 3 (1–7)
Ekizler et al. [54]	2019	Turkey	64	29.2 ± 6	NA	10 (15.6)	NA	NA
Elkayam et al. [55]	2019	USA	15	30 ± 7.3	1/6 (16.7)	NA	NA	6/10 (60)
Ersboll et al. [56]	2017	Denmark	61	31.7 ± 6.3	NA	33 (54)	5 (8.2)	29 (47.5)
Farhan et al. [57]	2021	Iraq	64	32.1 ± 6.8	NA	30/58 (51.7)	NA	49/61 (80.3)
Felker et al. [58]	2000	USA	42	29 ± 6	NA	11 (26)	5 (12)	22 (53)
Fett et al. [6]	2005	Haiti	98	Mean (range): 32.2 (16–50)	4 (4)	15 (15)	NA	74 (75.5)
Ford et al. [59]	2000	USA	11	28 ± 5	NA	3 (27.3)	0 (0)	NA
Gambahaya et al. [60]	2017	Zimbabwe	43	27.9 ± 6.0	NA	15 (34.9)	3 (7)	28 (65.1)
Goland et al. [61]	2011	USA	187	30 ± 6	NA	75/184 (41)	34/184 (18)	2.7 ± 2
Goland et al. [62]	2016	Israel	41	35 ± 6	5 (17)	10 (34)	7 (24)	2.3 ± 1.2
Goli et al. [63]	2021	Multinational	469	NA	NA	36.5%	NA	NA
Guldbrandt Hauge et al. [64]	2018	Denmark	61	NA	NA	33 (54.1)	NA	29 (47.5)
Gunderson et al. [9]	2011	USA	110	35+, n (%): 38 (34.5)	13 (11.8)	46 (41.8)	9 (8.2)	67 (60.9)
Gürkan et al. [65]	2017	Turkey	40	30 ± 5.9	NA	10 (25)	1 (2.5)	26 (65)
Haghikia et al. [66]	2013	Germany	115	34 ± 6	NA	50/112 (45)	17 (15)	2 (0–9)
Haghikia et al. [67]	2015	Germany	34	34 ± 5	4 (12)	NA	NA	Median (range): 1 (1–4)
Harper et al. [68]	2012	USA	85	35+, n (%): 27 (31.8%)	21/79 (26.6)	41/79 (52)	7/79 (8.9)	NA
Hasan et al. [69]	2010	Pakistan	32	32 ± 3	6(18.75)	NA	NA	23 (71.8)
Hassan et al. [70]	2019	Pakistan	60	30.1 ± 5.4	6 (10)	NA	15 (25)	86.7
Hilfiker-Kleiner et al. [71]	2017	Germany	63	NA	13 (20.6)	NA	NA	NA
Horgan et al. [72]	2013	Republic of Ireland	12	Mean (range): 34.7 (28–41)	9 (75)	NA	2 (17)	9 (75)
Horne et al. * [73]	2011	USA	71	GWA study: 29.8 ± 5.8 Replication study: 30.7 ± 6.6	18 (25.3)	NA	NA	NA
Huang et al. [74]	2010	China	82	29.5 ± 6.4	NA	11 (13.4)	NA	28 (34.1)
Huisman et al. [75]	2013	Netherlands	17	NA	NA	10 (58.8)	NA	NA
Irizarry et al. * [76]	2017	USA UPHS database	220	29.5 ± 6.6	NA	80/180 (44.4)	28 (13)	109 (49.5)
Isogai et al. [77]	2017	Japan	283	32.7 ± 7.5	48 (17)	53 (18.7)	NA	NA
Jackson et al. * [17]	2021	European PPCM Registry	735	no HDP: 30.3 ± 6.4 HDP: 31.7 ± 5.7 PE: 30.7 ± 6.6	184 (25)	283 (38.5)	33 (4.5)	362 (48.1)
Johansson et al. [78]	2021	Sweden	22	NA	14 (63.6)	NA	NA	NA
Kamiya et al. [79]	2011	Japan	102	HDP: 33.8 ± 4.2 no HDP: 31.9 ± 4.1	29(28.4)	42 (41.2)	15 (14.7)	1.62 ± 1.17 I 1.67 ± 0.78
Kao et al. [80]	2013	USA database	535	30+, n (%): 276 (51.6)	157 (29.3)	NA	60 (11.2)	NA
Karaye et al. * [7]	2020	Nigeria Registry	406	28.6 ± 7.2	64 (15.8)	NA	59 (14.5)	289 (71.2)
Karaye et al. [81]	2016	Nigeria	54	26.6 ± 6.7	NA	25 (46.3)	NA	NA
Kim et al. [82]	2020	Korea	21	32 ± 4.9	5 (23.8)	NA	8 (38.1)	7 (35)
Kolte et al. * [8]	2014	USA database	34219	30.3 ± 7	3867 (11.3)	5714 (16.7)	701 (2)	115 (0.3)
Laghari et al. [83]	2013	Pakistan	45	27.4 ± 6.1	4 (8.8)	NA	3 (6.6)	20 (44.4)
Lee et al. [84]	2018	Korea	795	32.1 ± 4.3	127 (16)	NA	52 (6.5)	356 (44.8)
Lewey et al. [20]	2020	USA	220	No HDP: 30.4 ± 5.8 HDP: 28 ± 7.2	NA	76/175 (43.4)	22 (10)	NA
Li et al. [85]	2016	China	71	28 ± 6	29 (40.1)	38 (54)	NA	14(19.7)
Lim et al. [86]	2013	China	11	32.3 ± 5.7	1 (9)	5 (45.5)	1 (9)	NA
Lindley et al. [87]	2017	USA	39	No PE: 29.3 ± 5.9 PE: 27.4 ± 7.4	17 (43.6)	NA	NA	No PE: 3.1 ± 1.9 PE: 2.6 ± 2.2
Ma et al. [88]	2019	China	80	29.2 ± 4.3	16 (20)	45 (56.2)	24 (30)	52 (65)
Mahowald et al. [89]	2019	USA	59	29.5 ± 6.8	13 (22)	21 (36)	NA	NA
Malhame et al. [90]	2019	Canada database	949	NA	283 (32.4)	358 (37.7)	NA	NA
Masoomi et al. [91]	2018	USA	568	Mean (95% CI): 30.0 (29.3– 30.6)	165 (29)	276 (48.6)	38 (6.7)	NA
McNamara et al. [92]	2015	North America	100	30 ± 6	NA	45 (45)	NA	2.2 ± 1.3
Midei et al. [93]	1990	USA	18	28 ± 1	NA	NA	NA	11 (61.1)
Moulig et al. [94]	2019	Germany	67	34 ± 5	9 (13.0)	15 (22.0)	13 (19.0)	1.7 ± 1
O’Connell et al. [95]	1986	USA	14	28.7 ± 5.7	NA	4 (29)	2 (14)	3 (21)
Osterman-Pla et al. [96]	2016	Puerto Rico	12	27 ± 8	6 (50.0)	NA	2 (16.7)	2.6 ± 1.6
Pandit et al. [97]	2009	India	9	28.5 ± 2.5	NA	1 (11.1)	NA	6 (66.7)
Patel et al. [98]	2016	Sweden	19	37 ± 5.8	13 (68)	14 (74)	4 (21)	NA
Perveen et al. [99]	2016	Pakistan	22	30, n (%): 12 (54.5)	2 (9.1)	18 (81.8)	2 (9.1)	8 (36.4)
Phan et al. [100]	2020	USA	333	Median (IQR): 33.2 (29.4– 36.9)	37 (11.1)	NA	NA	259 (77.8)
Pillarisetti et al. [19]	2014	USA	100	30 ± 6.5	36 (36)	69 (69)	NA	61 (61)
Prameswari et al. [101]	2018	Indonesia	96	Median (IQR): 30.5 (13)	NA	55 (57.3)	8 (8.3)	60 (62.5)
Prasad et al. [102]	2014	India	16	25.25	4 (25)	10 (62.5)	NA	7 (43.7)
Ravi Kiran et al. [103]	2021	India	43	25.4 ± 2.9	NA	8 (18.6)	NA	1.4 ± 0.8
Rosman et al. [104]	2017	USA	177	30.6 ± 5.5	56 (37.6)	87 (49.2)	NA	2.7 ± 1.7
Safirstein et al. [105]	2012	USA	55	31.7 ± 5.68	NA	23 (41.8)	8 (14.5)	28 (50.9)
Sagy et al. [106]	2017	Israel	42	30.8 ± 7	14 (33.3)		1 (2.4)	4.5 (1–7)
Saltzberg et al. [107]	2012	USA	107	31.2 ± 6.3	NA	42 (39)	15%	66.2%
Samonte et al. [108]	2013	Philippines	9	29.3 ± 8.7	7 (78)	NA	1 (6.2)	6 (66.6)
Shah et al. * [109]	2018	USA NRD database	6880	31.0 ± 6.8	1947 (28.3)	2243 (32.6)	1.7%	0.9%
Shani et al. [110]	2015	Israel	36	33.5 ± 6	NA	13 (38.9)	12 (33.3)	13 (36.1)
Sliwa et al. [111]	2018	Africa	269	28.6 ± 5.9	5 (1.8)	18 (6.7)	7 (3)	224 (83.3)
Sliwa et al. [112]	2006	USA	100	31.6 ± 6.6	NA	2(2)	NA	NA
Sugahara et al. [113]	2019	USA	90	Median (IQR): 31 (25–34)	NA	39 (43)	NA	NA
Sultan et al. [114]	2019	Pakistan	32	27.4 ± 5.8	1 (3.1)	10 (31.1)	NA	26 (81.2)
Tremblay-Gravel et al. [115]	2019	Canada	76	NA	20 (26.3)	31 (40.8)	8 (10.5)	40 (52.6)
Vettori et al. [116]	2011	Brazil	6	26.5 ± 7.1	3 (50)	NA	1 (16.7)	1 (16.7)
Whitehead et al. [117]	2003	USA	171	NA	NA	25 (15)	NA	NA
Witlin et al. [118]	1997	USA	9	33 ± 6.9	2 (22.2)	NA	NA	NA
Wu et al. [119]	2017	Taiwan	742	30.5 ± 5.7	146 (19.7)	NA	NA	55 (7.4)
Yang et al. [120]	2020	Korea	21	33 ± 5	6 (29)	16 (77)	5 (24)	5 (24)
Zhu et al. [121]	2021	China	60	30 ± 5	7 (11.7)	12 (20)	NA	36 (60)

* These studies may include some of the cohorts from other studies included in this systematic review; NIS, Nationwide Inpatient Sample; GWA, genome-wide association; HDP, hypertensive disorder of pregnancy; NA, not available; NRD, Nationwide Readmissions Database; UPHS, University of Pennsylvania Health System.

## Data Availability

The data that support the findings of this study are available on request from the corresponding author.

## Supplementary Materials

The following supporting information can be downloaded at https://www.mdpi.com/article/10.3390/jcm14051721/s1, Table S1: Quality of PPCM studies; Figure S1: GOSH graph effects of influential study on the pooled prevalence for (a) HDPs and (b) PE.
