# Supplementary material for: Hypertensive Disorders of Pregnancy and Peripartum Cardiomyopathy: A Meta-Analysis of Prevalence and Impact on Left Ventricular Function and Mortality
Source: J Clin Med. 2025 Mar 4;14(5):1721. doi: 10.3390/jcm14051721 (PMC11900926; doi:10.3390/jcm14051721)
Supplement: Supplementary file 1 [file jcm-14-01721-s001.zip › Supplemental Table S1.pdf]

**Table S1:** Quality of PPCM studies

| ID | Study | Selection | | | | Comparability | | Outcome | | | Overall Quality |
|---|---|---|---|---|---|---|---|---|---|---|---|
| | | HDP/PE group | Non-HDP/PE group | Assessment of pregnancy outcome | HDP/PE diagnosis | Controlled for GA/post-partum interval | Controlled for ≥1 other factor | Blinded assessment | Follow-up period | Complete follow up | |
| 1 | Abbas et al. | * | * | * | * | | * | | * | * | 7/9 |
| 3 | Afana et al. | * | * | * | | * | * | | * | * | 7/9 |
| 5 | Ata Akil et al. | | | * | | | | | * | * | 3/9 |
| 8 | Amos et al. | * | * | * | | | | | * | * | 5/9 |
| 9 | Arnaut et al. | * | * | * | | | * | | | | 4/6 |
| 11 | Aroney et al. | * | * | * | | | | | * | * | 5/9 |
| 12 | Arora et al. | * | * | * | | | | | | | 3/6 |
| 16 | Azibani et al. | * | * | * | | | * | | * | * | 6/9 |
| 18 | Barasa et al. | * | * | * | * | | | | * | * | 6/9 |
| 20 | Barbosa et al. | * | * | * | | | | | * | * | 5/9 |
| 21 | Behrens et al. | * | * | * | * | | | | * | * | 6/9 |
| 22 | Bernstein et al. | * | * | * | | | | | | | 3/6 |
| 25 | Binu et al. | * | * | * | | | | | * | * | 5/9 |
| 26 | Biteker et al. 2011 | * | * | * | | | | | * | * | 5/9 |
| 28 | Biteker et al. 2020 | * | * | * | | | | | | | 3/6 |
| 32 | Bortnick et al. | * | * | * | * | | | | | | 4/6 |
| 37 | Briasoulis et al. | | * | * | | | | | * | * | 4/9 |
| 40 | Carvalho et al. | * | * | * | | | | | | | 3/6 |
| 47 | Chang et al. | * | * | * | | | | | | | 3/6 |
| 322 | Chapa et al. | * | * | * | | | | | * | * | 5/9 |
| 51 | Cho et al. | * | * | * | | | | | | | 3/6 |
| 53 | Codsi et al. | * | * | * | | | | | * | * | 5/9 |

| ID | Study | Selection | | | | Comparability | | Outcome | | | Overall Quality |
|---|---|---|---|---|---|---|---|---|---|---|---|
| | | HDP/PE group | Non-HDP/PE group | Assessment of pregnancy outcome | HDP/PE diagnosis | Controlled for GA/post-partum interval | Controlled for ≥1 other factor | Blinded assessment | Follow-up period | Complete follow up | |
| 56 | Cuenza et al. | * | * | * | | | | | | | 3/6 |
| 104 | Cunningham et al. | * | * | * | | | | | | | 3/6 |
| 59 | Davis et al. 2019 | * | * | * | | | | | | | 3/6 |
| 60 | Davis et al. 2021 | * | * | * | | | | | | | 3/6 |
| 65 | Dhesi et al. | * | * | * | | | | | | | 3/6 |
| 70 | Douglass et al. | * | * | * | | | | | * | * | 5/9 |
| 73 | Duran et al. | | | * | | | | | * | * | 3/9 |
| 75 | Ekizler et al. | * | * | * | | | | | * | * | 5/9 |
| 78 | Elkayam et al. | * | * | * | | | | | | | 3/6 |
| 83 | Ersboll et al. | * | * | * | * | | | | * | * | 6/9 |
| 85 | Farhan et al. | * | * | * | | | | | | | 3/6 |
| 87 | Felker et al. | * | * | * | | | | | * | * | 5/9 |
| 93 | Fett et al. | * | * | * | | | | | * | * | 5/9 |
| 98 | Ford et al. | * | * | * | | | | | * | * | 5/9 |
| 102 | Gmabahaya et al. | * | * | * | | | | | | | 3/6 |
| 108 | Goland et al. 2011 | * | * | * | | | | | * | * | 5/9 |
| 111 | Goland et al. 2016 | * | * | * | | | | | * | * | 5/9 |
| 112 | Goli et al. | * | * | * | | | | | | | 3/6 |
| 114 | Gunderson et al. | * | * | * | | | | | | | 3/6 |
| 115 | Gurkan et al. | * | * | * | | | | | * | * | 5/9 |
| 118 | Haghikia et al. 2013 | * | * | * | | | | | * | | 5/9 |
| 119 | Haghikia et al. 2015 | * | * | * | | | | * | * | | 5/9 |
| 123 | Harper et al. | * | * | * | | | | | * | * | 5/9 |
| 124 | Hasan et al. 2010 | * | * | * | * | | | | | | 4/6 |
| 125 | Hassan et al. 2019 | * | * | * | | | | | | | 3/6 |

| ID | Study | Selection | | | | Comparability | | Outcome | | | Overall Quality |
|---|---|---|---|---|---|---|---|---|---|---|---|
| | | HDP/PE group | Non-HDP/PE group | Assessment of pregnancy outcome | HDP/PE diagnosis | Controlled for GA/post-partum interval | Controlled for ≥1 other factor | Blinded assessment | Follow-up period | Complete follow up | |
| 126 | Hauge et al. | * | * | * | * | | | | | | 4/6 |
| 128 | Hilfiker-Kleiner et al. | * | * | * | | | | | * | * | 5/9 |
| 133 | Horgan et al. | * | * | * | | | | | * | * | 5/9 |
| 134 | Horne et al. | * | * | * | | | | | * | * | 5/9 |
| 137 | Huang et al. | * | * | * | | | | | | | 3/6 |
| 139 | Huisman et al. | * | * | * | | | | | | | 3/6 |
| 141 | Irizarry et al. | * | * | * | | | | | | | 3/6 |
| 143 | Isogai et al. | * | * | * | | | | | | | 3/6 |
| 146 | Jackson et al. | * | * | * | | | | | * | | 4/9 |
| 147 | Johansson et al. | * | * | * | * | | | | | | 4/6 |
| 148 | Kamiya et al. | * | * | * | * | | | | * | * | 6/9 |
| 149 | Kao et al. | * | * | * | | | | | | | 3/6 |
| 151 | Karaye et al. 2016 | * | * | * | | | | | | | 3/6 |
| 154 | Karaye et al. 2020 | * | * | * | * | | | | | | 4/6 |
| 163 | Kim et al. | * | * | * | | | | | | | 3/6 |
| 165 | Kolte et al. | * | * | * | | | | | | | 3/6 |
| 170 | Laghari et al. | * | * | * | | | | | | | 3/6 |
| 175 | Lee et al. | * | * | * | | | | | | | 3/6 |
| 176 | Lewey et al. | * | * | * | | | | | * | * | 5/9 |
| 177 | Li et al. | * | * | * | * | | | | * | * | 6/9 |
| 181 | Lim et al. | * | * | * | | | | | * | | 4/9 |
| 183 | Lindley et al. | * | * | * | * | | | | * | | 5/9 |
| 193 | Ma et al. | * | * | * | | | | | | | 3/6 |
| 195 | Mahowald et al. | * | * | * | | | | | * | * | 5/9 |
| 197 | Malhame et al. | * | * | * | * | | | | | | 4/6 |

| ID | Study | Selection | | | | Comparability | | Outcome | | | Overall Quality |
|---|---|---|---|---|---|---|---|---|---|---|---|
| | | HDP/PE group | Non-HDP/PE group | Assessment of pregnancy outcome | HDP/PE diagnosis | Controlled for GA/post-partum interval | Controlled for ≥1 other factor | Blinded assessment | Follow-up period | Complete follow up | |
| 202 | Masoomi et al. | * | * | * | | | | | | | 3/6 |
| 206 | McNamara et al. | * | * | * | | | | | | | 3/6 |
| 209 | Midei et al. | * | * | * | | | | | | | 3/6 |
| 217 | Moulig et al. | * | * | * | | | | | | | 3/6 |
| 227 | O'Connell et al. | * | * | * | | | | | | | 3/6 |
| 228 | Osterman-Pla et al. | * | * | * | | | | | | | 3/6 |
| 229 | Pandit et al. | * | * | * | | | | | * | * | 5/9 |
| 230 | Patel et al. | * | * | * | | | | | | | 3/6 |
| 233 | Perveen et al. | * | * | * | | | | | | | 3/6 |
| 238 | Phan et al. | * | * | * | | | | | | | 3/6 |
| 240 | Pillarisetti et al. | * | * | * | | | | | * | * | 5/9 |
| 241 | Prameswari et al. | * | * | * | | | | | | | 3/6 |
| 242 | Prasad et al. | * | * | * | | | | | | | 3/6 |
| 248 | Ravi Kiran et al. | * | * | * | | | | | | | 3/6 |
| 256 | Rosman et al. | * | * | | | | | | | | 2/6 |
| 260 | Safirstein et al. | | | * | | | | | * | | 2/9 |
| 261 | Sagy et al. | * | * | * | | | | | * | * | 5/9 |
| 263 | Saltzberg et al. | * | * | * | | | | | | | 3/6 |
| 264 | Samonte et al. | * | * | * | | | | | * | * | 5/9 |
| 268 | Shah et al. | * | * | * | | | | | * | * | 5/9 |
| 270 | Shani et al. | * | * | * | | | | | | | 3/6 |
| 279 | Sliwa et al. 2018 | * | * | * | | | | | | | 3/6 |
| 281 | Sliwa et al. 2006 | * | * | * | | | | | | | 3/6 |
| 292 | Sugahara et al. | * | * | * | | | | | | | 3/6 |
| 293 | Sultan et al. | * | * | * | | | | | | | 3/6 |

| ID | Study | Selection | | | | Comparability | | Outcome | | | Overall Quality |
|---|---|---|---|---|---|---|---|---|---|---|---|
| | | HDP/PE group | Non-HDP/PE group | Assessment of pregnancy outcome | HDP/PE diagnosis | Controlled for GA/post-partum interval | Controlled for ≥1 other factor | Blinded assessment | Follow-up period | Complete follow up | |
| 300 | Tremblay-Gravel et al. | * | * | * | | | | | | | 3/6 |
| 304 | Vettori et al. | * | * | * | | | | | * | * | 5/9 |
| 309 | Whitehead et al. | * | * | * | | | | | * | * | 5/9 |
| 311 | Witlin et al. | * | * | * | | | | | | | 3/6 |
| 315 | Wu et al. | * | * | * | | | | | | | 3/6 |
| 318 | Yang et al. | * | * | * | | | | | | | 3/6 |
| 321 | Zhu et al. | * | * | * | | | | | * | * | 5/9 |
