# Supplementary figures and images for: Hypertensive Disorders of Pregnancy and Peripartum Cardiomyopathy: A Meta-Analysis of Prevalence and Impact on Left Ventricular Function and Mortality
Source: J Clin Med. 2025 Mar 4;14(5):1721. doi: 10.3390/jcm14051721 (PMC11900926; doi:10.3390/jcm14051721)

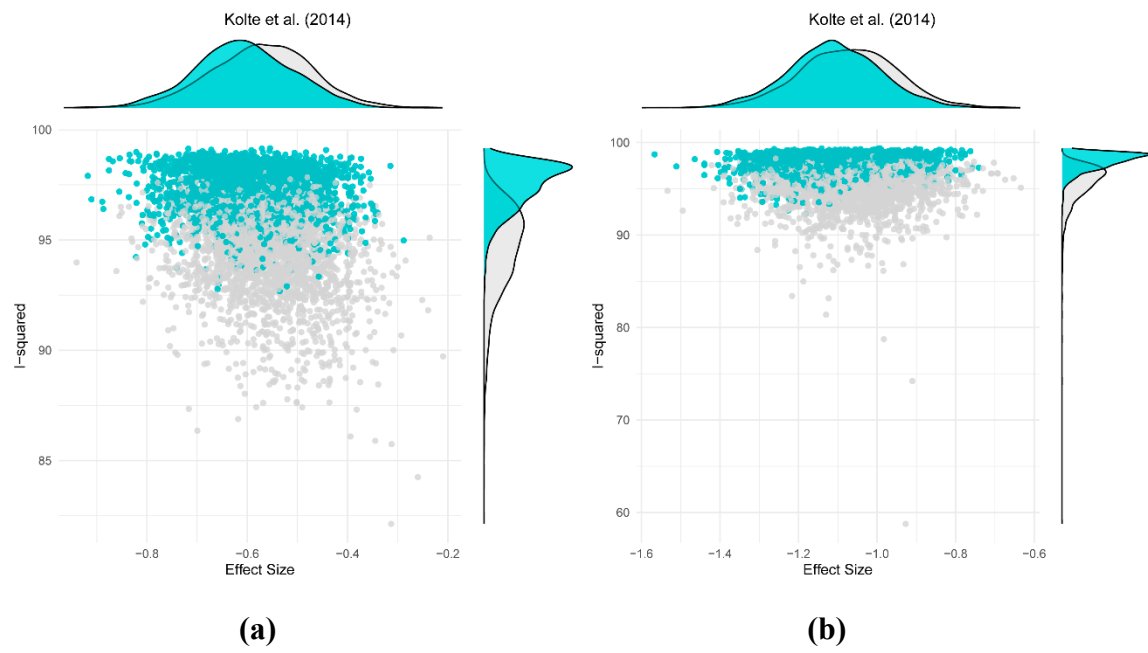

**Figure S1.** GOSH graph effects of influential study on the pooled prevalence for (a) HDP and (b) PE

Supplement: Supplementary file 1 [file jcm-14-01721-s001.zip › Supplemental Figure S1a,b.pdf]
